# Study on Control of Polymeric Architecture of Sulfonated Hydrocarbon-Based Polymers for High-Performance Polymer Electrolyte Membranes in Fuel Cell Applications

**DOI:** 10.3390/polym13203520

**Published:** 2021-10-13

**Authors:** Mijeong Kim, Hansol Ko, Sang Yong Nam, Kihyun Kim

**Affiliations:** Department of Materials Engineering and Convergence Technology, Gyeongsang National University, Jinju 52828, Korea; mijeong@gnu.ac.kr (M.K.); hansol.pmc@gnu.ac.kr (H.K.); walden@gnu.ac.kr (S.Y.N.)

**Keywords:** polymer electrolyte membrane fuel cell, perfluorinated sulfonic acid ionomer, sulfonated hydrocarbon polymer, phase-separation

## Abstract

Polymer electrolyte membrane fuel cell (PEMFC) is an eco-friendly energy conversion device that can convert chemical energy into electrical energy without emission of harmful oxidants such as nitrogen oxides (NO_x_) and/or sulfur oxides (SO_x_) during operation. Nafion^®^, a representative perfluorinated sulfonic acid (PFSA) ionomer-based membrane, is generally incorporated in fuel cell systems as a polymer electrolyte membrane (PEM). Since the PFSA ionomers are composed of flexible hydrophobic main backbones and hydrophilic side chains with proton-conducting groups, the resulting membranes are found to have high proton conductivity due to the distinct phase-separated structure between hydrophilic and hydrophobic domains. However, PFSA ionomer-based membranes have some drawbacks, including high cost, low glass transition temperatures and emission of environmental pollutants (e.g., HF) during degradation. Hydrocarbon-based PEMs composed of aromatic backbones with proton-conducting hydrophilic groups have been actively studied as substitutes. However, the main problem with the hydrocarbon-based PEMs is the relatively low proton-conducting behavior compared to the PFSA ionomer-based membranes due to the difficulties associated with the formation of well-defined phase-separated structures between the hydrophilic and hydrophobic domains. This study focused on the structural engineering of sulfonated hydrocarbon polymers to develop hydrocarbon-based PEMs that exhibit outstanding proton conductivity for practical fuel cell applications.

## 1. Introduction

Recently, interest in eco-friendly alternative energy has increased due to the depletion of fossil fuels and environmental pollution, many countries are making great efforts to develop renewable energy technologies that can replace fossil fuels [1]. In particular, studies that utilize hydrogen as an energy source are being actively conducted [2,3,4]. As the most abundant element in the universe, there are clearly no concerns with its depletion. The fuel cell is an eco-friendly energy conversion device that can generate electrical energy by the electrochemical reaction between the fuel supplied (mainly hydrogen) and oxidizers [5]. Unlike conventional power generation technologies, fuel cells can efficiently utilize hydrocarbon-based energy sources without emission of noise or vibration. Furthermore, since combustion process is not required, harmful substances such as nitrogen oxides (NO_x_) and sulfur oxides (SO_x_) are not generated during operation [6]. As shown in Table 1, fuel cells can be classified according to the type of electrolyte they use, and include alkali fuel cells (AFCs), molten carbonate fuel cells (MCFCs), solid oxide fuel cells (SOFCs) and polymer electrolyte membrane fuel cells (PEMFCs) [7,8,9,10].

**Figure 1:**
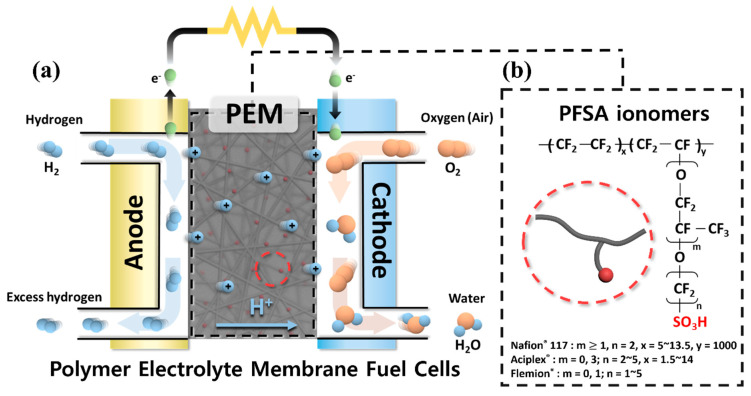
(**a**) Schematic diagram of polymer electrolyte membrane fuel cells. (**b**) Chemical structures of perfluorosulfonic acid ionomers.

In particular, PEMFCs have been studied intensively due to their high energy efficiency (even at low temperature) and the numerous applications they have proven suitable for, such as electric vehicles and portable devices (Figure 1a). Among the various components that constitute PEMFCs, polymer electrolyte membranes (PEMs) that provide a pathway for proton transport and prevent the permeation of the supplied fuels and electrons are regarded as the key component [11]. It is well known that protons can be transported from the anode to the cathode through the interconnected ionic clusters formed by phase separation between hydrophilic–hydrophobic domains in PEMs [12,13]. Therefore, the polymers used in PEMs should have hydrophobic segments that can maintain the membrane stability and hydrophilic segments containing fixed anionic groups that can transport protons [14]. Due to the high selectivity of PEMs regarding the target molecules, the possible wide application of PEMs as solid or gel-type electrolytes in various energy conversion and storage devices such as solar cells and secondary battery systems have also been recently studied [15,16,17,18,19,20,21,22]. In addition, considerable experimental and theoretical studies have also been performed to elucidate the effective diffusivity for the target molecules in PEMs used in such energy devices [23,24].

**Figure 2:**
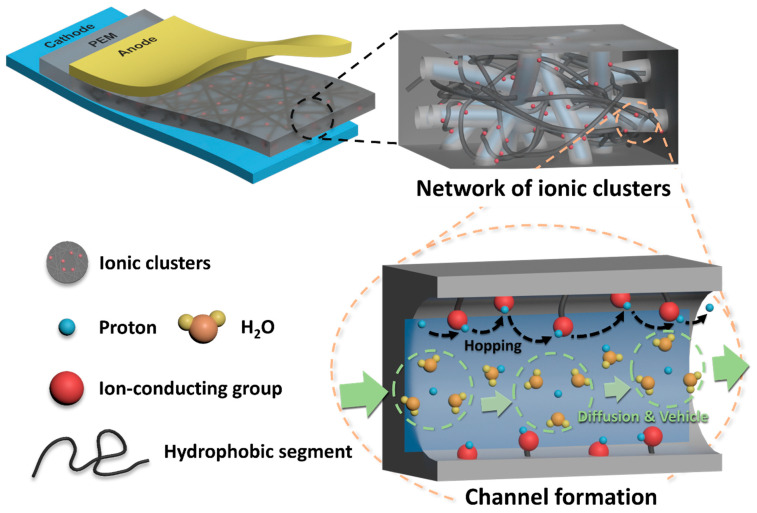
Schematic diagram of ionic clusters formed in polymer electrolyte membranes.

After decades of research, perfluorosulfonic acid (PFSA) ionomer-based PEMs such as Nafion^®^, Aciplex^®^ and Flemion^®^ have been developed and applied in commercialized PEMFC vehicle systems including the Toyota Mirai (2015), the Honda Clarity (2016) and the Hyundai Nexo (2018) [25]. This is because the PFSA ionomers, shown in Figure 1b, are composed of a hydrophobic perfluoro backbone and the flexible hydrophilic side chains containing sulfonic acid groups, the interconnected ionic clusters formed by the phase separation between the hydrophobic and hydrophilic domains are well developed (Figure 2). Therefore, the PFSA ionomer-based PEMs can maintain high proton conductivity despite their low ion exchange capacity (IEC) and reveal outstanding physical stability even under highly humidified operating conditions. In addition, since the PFSA ionomers are mainly composed of C-F bond having a strong bonding energy (485 kJ/mol at 273 K), the corresponding PEMs exhibit outstanding chemical stability under the harsh operating conditions of PEMFCs [26,27]. Nevertheless, the use of PFSA ionomer-based PEMs still has inherent drawbacks, which include limited operating temperatures due to their low glass transition temperature, expensive manufacturing costs due to the complicated synthetic process and environmental problems caused by production of toxic pollutants (e.g., HF) during the disposal process [28]. These drawbacks have prompted the development of alternative PEMs via different strategies which have included modifying the PFSA-ionomer structure, introducing inorganic/organic composite materials and developing sulfonated hydrocarbon-based polymers [11,29,30].

The sulfonated hydrocarbon polymers (SHPs) have been intensively studied due to their outstanding thermal stability, high mechanical strength as well as low fuel cross-over [31,32,33,34]. In addition, it is possible to develop PEMs at low cost due to the relatively convenient synthetic process compared to that of the PFSA ionomers. Moreover, various types of SHPs with different IECs can be obtained by incorporating diverse monomers and/or by post-modification of intermediates [35,36,37]. Representative SHP structures including sulfonated poly(arylene ether sulfone) (SPAES) [38,39,40,41,42,43], sulfonated poly(ether ether ketone) (SPEEK) [44,45], sulfonated poly(phenylene oxide) (SPPO) and sulfonated polyimide (SPI) [46,47] are shown in Table 2. Although PEMs with these polymers have been studied as alternatives to the PFSA ionomer-based PEMs due to the advantages described, the proton conductivity of the SHP-based PEMs is generally lower than that of the PFSA ionomer-based PEMs, because the interconnected hydrophilic channels are not as well developed as PFSA ionomer-based PEMs [48]. Typically, SHPs having a high degree of sulfonation (DS) can form large hydrophilic domains, resulting in high proton conductivity. However, when the DS of SHPs is high enough to reach a comparable proton conductivity as that of PFSA ionomer-based PEMs, they do not maintain the necessary high physicochemical stability for PEMFC operation [30,49,50]. To improve the proton conductivity of SHP-based PEMs without the deterioration in physicochemical stability, structural engineering of the SHPs has been conducted to form distinct phase-separated structures of the hydrophilic and hydrophobic domains, similar to those of PFSA ionomers [51,52,53]. It is generally known that control of hydrophilic and hydrophobic segments within SHPs can be achieved by the preparation of block, graft/comb-shaped and densely sulfonated copolymers [54,55,56]. Therefore, this study reports on recent research trends related to the development of SHP-based PEMs showing high performances in PEMFCs by pursuing rational design strategies for the copolymer architectures.

## 2. Structural Engineering of Sulfonated Hydrocarbon Polymers for PEMFC Applications

### 2.1. Block Copolymer-Based PEMs

**Figure 3:**
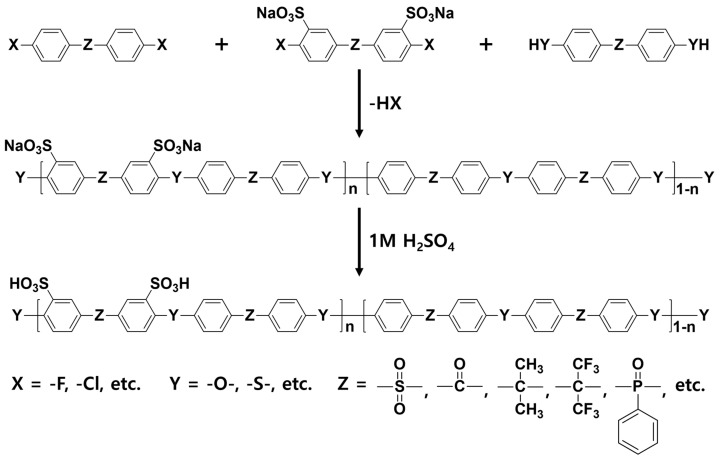
Synthetic procedures of sulfonated hydrocarbon-based random copolymers.

In general, sulfonated hydrocarbon polymers synthesized via the nucleophilic aromatic substitution reaction between dihalo monomers with or without sulfonic acid groups and difunctional monomers with nucleophiles (e.g., dihydroxy and dithiol) are composed of randomly distributed hydrophilic and hydrophobic moieties due to the random distribution of hydrophilic sulfonic acid groups (Figure 3). Therefore, the PEMs prepared by random copolymers usually exhibit lower proton conductivity compared to the PFSA-based PEMs, especially at low relative humidity (% RH) conditions, due to the low hydrophilic/hydrophobic phase separation behavior which forms the small ion-conducting channels [57,58]. Therefore, structural engineering of SHP-based polymer beginning with the synthetic process is highly required to control the nano-phase structures of the resulting PEMs.

It is well known that block copolymers synthesized by assembling the hydrophilic and hydrophobic oligomers forming di-block, tri-block and multi-blocks can effectively control the nano-phase structure and facilitate the distinct phase separation characteristics of the hydrophilic/hydrophobic moieties [59,60,61,62]. Therefore, the block copolymer-based PEMs are able to exhibit outstanding proton conductivity even under low RH conditions and greatly reduce the conductivity dependence on temperature and humidity changes. In addition, due to the well-defined phase-separated structure, the dimensional and chemical stabilities of the block copolymer-based PEMs can be improved compared to those of the random copolymer-based PEMs having similar ion exchange capacities (IECs).

**Figure 4:**
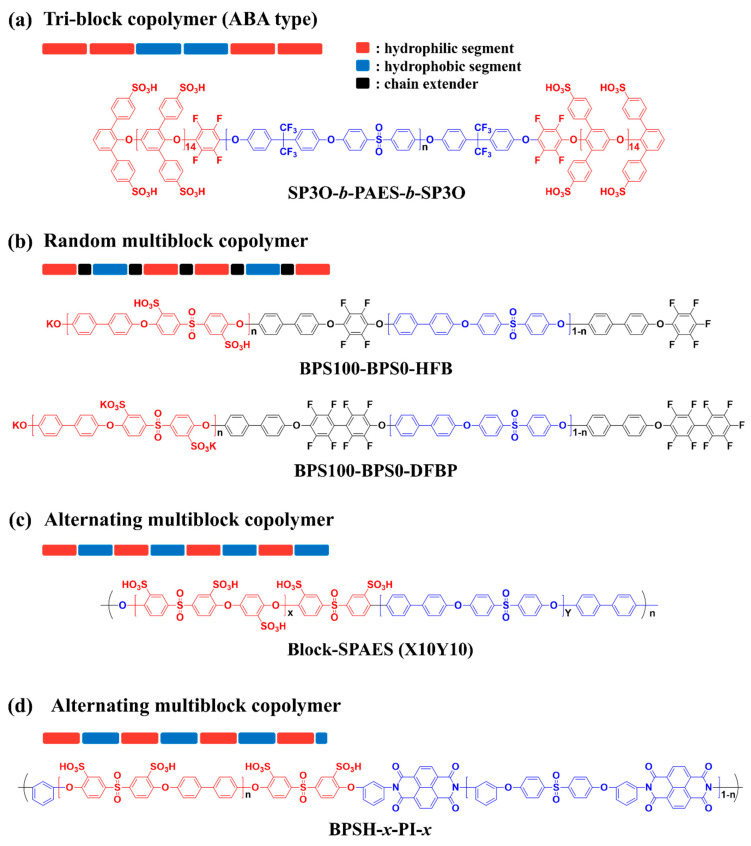
Representative chemical structures of sulfonated hydrocarbon-based block copolymers [63,64,67,68].

Representatively, Michael D. Guiver et al. reported development of a PEM with ‘ABA’ type tri-block copolymer (SP3O-*b*-PAES-*b*-SP3O) composed of sulfonated poly(2,6-diphenyl-1,4-phenylene oxide)s (SP3O) as a hydrophilic ‘A’ block and poly(arylene ether sulfone) (PAES) as a hydrophobic ‘B’ block (Figure 4a) [63]. Although the IEC value (0.97 meq g^−1^) of the SP3O-*b*-PAES-*b*-SP3O membrane was found to be smaller than those of other reported hydrocarbon-based PEMs, showing the values from 1.50 to 2.34 meq g^−1^, the size of the ionic clusters observed by atomic force microscopy (AFM) and transmission electron microscopy (TEM) were 15 nm and 5–10 nm, respectively, and these are comparable to or larger than those of Nafion^®^ 112. Therefore, the proton conductivities of the SP3O-*b*-PAES-*b*-SP3O membrane are comparable to those of Nafion^®^ 112 under low humidity conditions from 30 to 50% RH. 

James E. McGrath et al. developed multi-block copolymers using phenoxide-terminated sulfonated poly(arylene ether sulfone) (BPS100) with different chain lengths as the hydrophilic oligomers and poly(arylene ether sulfone) (BPS0) with different chain lengths as the hydrophobic. Two different types of block copolymers were prepared by incorporating different types of perfluoroaryl chain extenders such as hexafluorobenzene (HFB) and decafluorobiphenyl (DFBP), as shown in Figure 4b [64]. The resulting multi-block copolymer-based membranes revealed well-defined ion-conducting channels with distinct phase separation between hydrophilic and hydrophobic domains. In addition, the membrane properties including ion conductivity could be adjusted by changing the hydrophilic/hydrophobic block length (e.g., 5k–5k, 10k–10k, 15k–15k, etc.) [65,66]. Furthermore, it was noted that membranes formed using DFBP as the chain extender, BPS100-BPS0-DFBP, showed a more distinct phase-separated structure than with HFB as the chain extender (BPS100-BPS0-HFB). This occurs due to the higher content of fluorine moieties in DFBP, which increases the hydrophobicity of the BPS0 oligomers and the acidity of sulfonic acid groups in the hydrophilic BPS100. This observation indicates that the molecular structure of the chain extender affects the properties of the resulting multi-block copolymer-based PEMs. 

The incorporation of highly reactive reagents such as HFB and DFBP as chain extenders can efficiently increase the molecular weight of block copolymer by increasing the reaction rate of hydrophilic and hydrophobic segments. However, the structure of the resulting block copolymer is usually random multiblock copolymers due to the same telechelic functionality of the hydrophilic and hydrophobic oligomers. Recently, Byungchan Bae et al. reported detailed synthetic strategies for the development of alternating multi-block copolymer with high-molecular weight by using hydrophilic and hydrophobic segments with different telechelic functional groups. A transparent and flexible PEM with IEC value of 2.9 meq g^−1^ could be obtained using this alternating multi-block copolymer (X10-Y10), as shown in Figure 4c [67]. Interestingly, the proton conductivity of this block copolymer PEM is four times higher than that of PFSA ionomer-based PEMs such as Nafion^®^ and Aquivion^®^ at 80 °C and RH 90%. As well, the conductivity is also comparable to that of the PFSA ionomer-based PEMs even under low RH conditions (≤50%). The distinct phase-separated structure confirmed by TEM as well as the large IEC value of the alternating multi-block copolymer membrane could support its outstanding proton conductivity. 

A multi-block copolymer incorporating polyimide (PI) moieties into the main chain was developed by James E. McGrath et al. (Figure 4d) [68]. As PI is vulnerable to acidic conditions, a modified PI was adopted to improve the hydrolysis resistance of the PI-based PEMs under fuel cell operating conditions. Multi-block copolymers, utilizing sulfonated poly(arylene ether sulfone)-*b*-polyimide (BPSH-*x*-PI-*x*, where *x* indicated the chain length of the hydrophilic and hydrophobic oligomers, respectively), were synthesized via imidization using hydrophilic BPSH oligomers with amine functional end groups and hydrophobic PI oligomers with anhydride end groups. The experimental IEC of the BPSH-*x*-PI-*x* membrane was found to diminish as the chain lengths of the hydrophilic and hydrophobic moieties increased, although all IEC values showed a similar range from 1.22 to 1.65 (meq g^−1^). AFM analysis of the BPSH-*x*-PI-*x* membranes indicated that the connectivity and size of the ion-conducting channels were better formed as the chain length of each block increased. Accordingly, water uptake of the membranes increased as the length of each block increased, but it decreased sharply when hydrophilic/hydrophobic chain lengths were each over 20 k (i.e., BPSH-*20*-PI-*20*) due to the enhanced phase separation as well as entanglement between intermolecular chains. In terms of proton conductivity, the BPSH-*15*-PI-*15* membrane possessed moderate ion-conducting channels, and the largest water absorption behavior exhibited the largest conductivity values among the samples and these values were larger than those of Nafion^®^ 112 at 80 °C. Based on these results, it can be concluded that optimizing chain lengths of the hydrophilic/hydrophobic blocks can effectively engineer the ion-conducting channels and thereby control membrane properties [65,69]. Due to the outstanding proton conductivity of the block copolymer-based PEMs by constructing the well-defined phase-separated structures, the MEAs prepared with these PEMs have been tried to apply in fuel cell vehicles operating at low RH conditions. The PEM properties of the above-described block copolymer-based PEMs including IEC, water uptake and proton conductivity are summarized in Table 3.

### 2.2. Graft/Comb-Shaped Copolymer-Based PEMs

The graft or comb-shaped copolymers used in PEMFCs are usually composed of physically and chemically stable hydrophobic main chains and proton-conducting hydrophilic side chains. By geometric separation of the structural units of the copolymers that are responsible for stability and ion conductivity, the PEMs with graft copolymers were found to have a distinct phase-separated structure between hydrophilic/hydrophobic domains and reveal better proton conductivity, oxidative and dimensional stabilities compared to their linear counterparts of similar compositions [62,70,71]. Notably, some studies have reported that comb-shaped copolymer-based membranes show better PEM properties including proton conductivity and lower water absorption behavior, resulting in smaller dimensional change than block copolymer-based PEMs with similar compositions [61,72]. 

**Figure 5:**
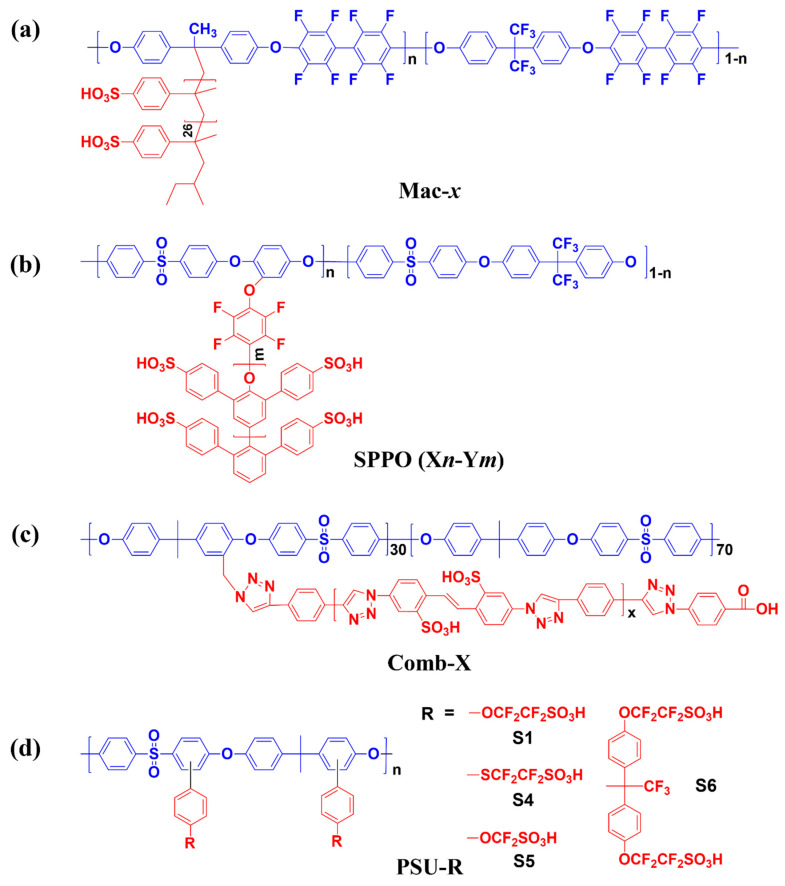
Representative chemical structures of sulfonated hydrocarbon-based graft/comb-shaped copolymers. (**a**) Mac-*x*, (**b**) SPPO (X*n*-Y*m*), (**c**) Comb-X and (**d**) PSU-R [49,73,80,82].

Jianfu Ding et al. reported graft copolymers composed of partially fluorinated poly(arylene ether) main chains as a rigid hydrophobic main backbone and oligomeric sulfonated polystyrene as flexible hydrophilic side chains, as shown in Figure 5a [73]. In this study, weight fractions (wt %) of the side to the main chains in the graft copolymers were 19, 25 and 38% and designated as 1a, 2a and 3a, respectively. Based on cross-sectional TEM images, as the hydrophilic side chain moieties increased, the size of the ionic clusters inducing the distinct phase-separated structure became larger. Accordingly, the 3a membrane having the largest content of sulfonated polystyrene side chains showed better proton conductivities than Nafion^®^ 117 measured in water under the entire range of temperatures from 20 to 80 °C. It was also demonstrated that the 2a membrane showed a smaller dimensional change value than Nafion^®^ 117, although it had a larger IEC (1.40 meq g^−1^) than Nafion^®^ 117 (0.96 meq g^−1^). Moreover, the 1a membrane having a similar IEC of Nafion^®^ 117 showed half the dimensional change in the longitudinal direction compared to Nafion^®^ 117, which is significantly lower than previously reported hydrocarbon-based PEMs [74]. Overall, it is demonstrated that membranes composed of graft copolymer with poly(arylene ether) main chains and oligomeric sulfonated polystyrene side chains effectively controlled the water absorption behavior without a deterioration of proton conductivity. However, preliminary cell performance tests using the membrane electrode assemblies (MEAs) with these membranes revealed a rapid drop in cell voltages with increases in the current density, due to chemical degradation of the highly reactive benzylic position of the polystyrene [75,76,77,78]. Therefore, in addition to the geometric separation of hydrophilic and hydrophobic regions of the graft copolymer, the authors emphasized that one of the most important aspects in future development of graft copolymer-based PEMs was that the chemical composition of the side chains should not contain chemically vulnerable bonds. This will be necessary to ensure long-term durability for practical fuel cell operation [65,79]. 

Michael D. Guiver et al. developed graft copolymers using sulfonated poly(phenylene oxide) (SPPO) as a hydrophilic aromatic side chain to overcome the instability of aliphatic hydrocarbon and polystyrene type of side chains in graft copolymers for PEMs, in order to examine whether they showed excellent chemical and thermal stabilities, and high mechanical strength [80]. The graft copolymers (n5-m6, n5-m9, n3-m14, n5-m14) composed of poly(arylene ether sulfone) (PAES) as the main chain and SPPO as side chain were prepared by adjusting the ratio of the repeating units of PAES (n) and the length of SPPO side chains (m) (Figure 5b). Based on TEM images of the membranes, the sizes of ionic clusters were highly dependent on the length of the hydrophilic side chain. The sizes of the ionic clusters and IEC of the n5-m6 membrane (showing the smallest ionic clusters) were 3–5 nm and 0.92 meq g^−1^, respectively, which are similar to those of Nafion^®^ 112 (5–8 nm, 0.90 meq g^−1^). In this study, it was noteworthy that all the graft copolymer membranes formed well-defined ion-conducting channels via through-plane directionality, thereby exceptionally small dimensional change was observed in the in-plane direction compared to the thickness direction. As all membranes showed comparable or larger IECs and ionic clusters, the proton conductivities of all membranes were larger than Nafion^®^ 112 at 90 °C across the entire range of RH. 

Recently, Jong-Chan Lee et al. reported comb-shaped polysulfone copolymers containing sulfonated polytriazole side chains for high-performance PEMs in fuel cell applications [49]. The comb-shaped copolymers with different side chain lengths (comb-X, where X indicated the side chain length) were prepared via azide-alkyne click reaction of polysulfone with azidomethyl groups and sulfonated polytriazoles with a terminal ethynyl group (Figure 5c). It was confirmed that the comb-X membranes exhibited better mechanical strength than the linear polysulfone membrane (i.e., main chain-based membrane) due to the presence of intra/inter molecular acid-base interactions between the sulfonic acid and triazole groups in sulfonated polytriazoles [81]. Moreover, the comb-X membranes exhibited superior physical and oxidative stabilities as well as proton conductivity compared to the random-type sulfonated poly(arylene ether sulfone) (SPAES) membranes that have IECs similar to those of the comb-X membranes, due to the distinct phase-separated structure formed by the comb-shaped architectures. Subsequently, the fuel cell performances of the MEAs with comb-X membranes were superior to those with the SPAES membranes and better than that with the Nafion^®^ 212 membrane at various operating conditions. The authors demonstrated that the rational design of comb-shaped architectures possibly forming additional interactions between the side chains can improve both the physicochemical stabilities and the electrochemical properties of the SHP-based PEMs. 

Chulsung Bae et al. elucidated synthetic strategies for graft copolymers taking into consideration the effect of acidity of the sulfonic acid groups in the side chains and the optimal lengths for the geometric separation between the main and side chains [82]. As shown in Figure 5d, graft copolymers were synthesized using different types of super acidic fluorosulfonic acid groups (R) with different connecting groups, chain lengths and mole fractions. Membranes prepared using S5 with the shortest chain length showed the smallest ionic cluster size (1–2 nm) and proton conductivity among the samples due to the smallest electron-withdrawing effect. As such, it is difficult to form phase-separated structures between the hydrophobic main and hydrophilic side chains. In terms of electron-withdrawing effects, S1 and S4 are more acidic than S5 due to the additional -CF_2_ bonds, and therefore, showed better proton conductivity than the S5 membrane. Comparing S4 and S1 membranes, S4 with sulfur bonds (-SCF_2_-) having larger orbital and polarity than S1 with oxygen bonds (-OCF_2_-) leads to better conductivity and water absorption behavior. Interestingly, S6 membrane with the longest and largest side chain was found to develop distinct phase-separated structures with 3 nm ionic clusters due to the geometric separation by physical distance between main and side chains, resulting in the highest proton conductivity among the samples and superior to that of Nafion^®^ at over 50% RH. This study demonstrated that the outstanding proton conductivity of the comb-shaped/graft copolymer-based PEMs could be achieved by considering both the acidity of the attached sulfonic acid groups and the physical separation of hydrophobic and hydrophilic moieties to construct distinct phase-separated structures [66]. Due to the outstanding proton conductivity as well as physical stability, the graft/comb-shaped copolymer-based PEMs have been actively studied to instead of PFSA ionomer-based PEMs. The preliminary PEMFC tests employing the graft/comb-shaped copolymer-based PEMs have been tried to apply in electrical transport and portable device applications. The PEM properties of the above-described sulfonated hydrocarbon-based graft/comb-shaped copolymer-based PEMs including IEC, water uptake and proton conductivity are summarized in Table 4.

### 2.3. Densely Sulfonated Copolymer-Based PEMs

One of the effective strategies for development of PEMs with the outstanding proton conductivity under low RH conditions is the preparation of densely sulfonated copolymer having a large number of sulfonic acid groups located at a specific portion of copolymers [83,84]. Since the sulfonic acid groups are concentrated locally, well-defined phase-separated structures inducing comparable or better proton conductivity than PFSA-based ionomer PEMs have been reported with densely sulfonated copolymer-based PEMs [54,85,86,87]. Meanwhile, the degree of sulfonation and the bulkiness of the sulfonic acid moieties should be considered during structure engineering, because the water absorption behavior including water uptake and dimensional change in the corresponding PEMs is highly affected by their sulfonated portion of copolymers [88,89,90,91]. 

**Figure 6:**
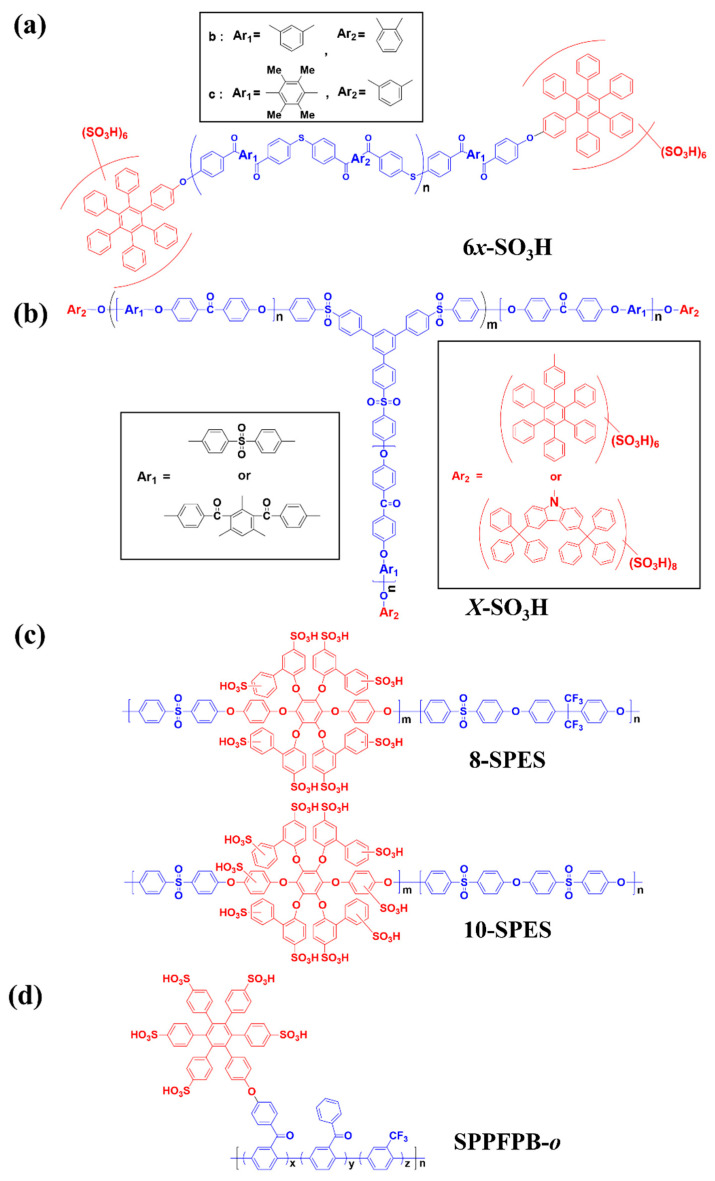
Representative chemical structures of densely sulfonated hydrocarbon-based copolymers. (**a**) 6*x*-SO_3_H, (**b**) *X*-SO*_3_*H, (**c**) 8-SPES, 10-SPES and (**d**) SPPFPB-*o* [92,93,94,95,96,99].

Allan S. Hay et al. reported densely sulfonated copolymers containing six sulfonic acid groups at the end group of the main chain (Figure 6a) [92]. Since the main chain was composed of poly(sulfide ketone) (PSK)-based rigid aromatic polymer, the resulting PEMs were found to have low methanol permeability. Furthermore, due to the electron-withdrawing effect of the ketone groups, the copolymer PEMs showed outstanding proton conductivity, possibly from the increase in the acidity of the sulfonic acid groups, in addition to the phased separated structure between hydrophilic and hydrophobic moieties. Although the densely sulfonated copolymer membranes (termed 6b-SO_3_H and 6c-SO_3_H according to the structure of Ar_1_ and Ar_2_ in the main chain of PSK) revealed relatively low IEC values (0.48 meq g^−1^ and 0.47 meq g^−1^, respectively) compared to other SHP-based PEMs, the proton conductivities of these membranes were superior to other reported PEMs with similar IECs due to the well-defined phase-separated structures. However, the proton conductivities of the 6b-SO_3_H and 6c-SO_3_H are lower than those of the PFSA-based PEMs due to the two-times-lower IEC values of the copolymer PEMs. To improve the IEC and proton conductivity of these sulfonated PEMs, follow-up studies were conducted by the same group. Figure 6b shows branched poly(ether ketone) (PEK) copolymers with three ends having multiple sulfonic acid groups [93,94]. The end-capping reagents of the PEK are hexaphenylbenzene and 3,6-ditrityl-9*H*-carbazole, respectively; thus, the maximum numbers of sulfonic acid groups that can be introduced in these branched PEKs are 18 and 24, respectively. The larger IEC and proton conductivity values observed in the PEM with the 3,6-ditrityl-9*H*-carbazole were clearly due to the higher sulfonic acid group content. For example, the maximum IEC and proton conductivity of the PEM containing PEK with 3,6-ditrityl-9*H*-carbazole were 1.25 meq g^−1^ and 95 mS cm^−1^ (at 30 °C and 100% RH), respectively, while those with hexaphenylbenzene achieved 1.05 meq g^−1^ and 91 mS cm^−1^ respectively. Although the size of ionic clusters observed in both membranes by TEM were smaller (2–3 nm) than that of Nafion^®^ 117 (5–8 nm), distinct phase-separated structures similar to that of Nafion^®^ 117 could be observed. Therefore, the better proton conductivities of the PEK-based PEMs than Nafion^®^ 117 are obtained due to the well-developed phase-separated structures, in addition to their larger IECs. 

Mitsuru Ueda et al. synthesized sulfonated poly(ether sulfone) (SPES) copolymers having eight densely located sulfonic acid groups in the bulky moieties (termed 8-SPES) by the general nucleophilic aromatic substitution of 4,4′-dichlorodiphenylsulfone, 1,2,4,5-tetrakis([1,1′-biphenyl]2-oxy)-3,6-bis(4-hydroxyphenoxy)benzene and 2,2-bis(4-hydroxyphenyl)hexafluoropropane followed by sulfonation [95]. The resulting copolymer structure is shown in Figure 6c. Due to the larger polarity difference between fluorine-containing hydrophobic and sulfonic-acid-containing hydrophilic bulky segments, distinct phase-separated structures as well as outstanding proton conductivity of membranes could be demonstrated. For example, the proton conductivities of membranes prepared with the SPES-based copolymers having molar ratios of hydrophilic:hydrophobic segments of 17:83 and 20:80, respectively, were found to be comparable to or better than those of Nafion^®^ 117 at 80 °C. The ionic cluster size of the 20:80 SPES-based PEM was around 5 nm, which is also similar to that of the Nafion^®^ 117. To reduce the RH dependency of the SPES-based PEMs, a follow-up study was performed by the same group [96]. The molecular structure of the advanced SPES copolymers having ten sulfonic acid groups in the repeating unit of the hydrophilic bulky moieties (10-SPES) is also shown in Figure 6c. Since the polarity difference between the hydrophilic and hydrophobic repeating units of 10-SPES is larger than that of 8-SPES due to the higher content of sulfonic acid groups, the 10-SPES membrane showed better physical stability and mechanical strength with similar IEC values. For example, the 8-SPES membrane with an IEC of 2.4 meq g^−1^ was clearly dissolved in water, while the 10-SPES membrane with the same IEC value maintained its dimension due to the relatively larger content of the hydrophobic repeating unit. Furthermore, the 10-SPES membrane showed better conductivity than the 8-SPES membrane due to the enhanced polarity difference resulting in a more distinct phase-separated structure [97,98]. 

Recently, Young Taik Hong et al. reported densely sulfonated poly(*p*-phenylene)-based copolymers with highly sulfonated multi-phenyl pendant groups (Figure 6d) [99]. These membranes (termed SPPFPB-*o_,_* where *o* indicates the (y + z)/x molar ratio, with x = hydrophilic portion, y = hydrophobic portion of 2,5-dichlorobenzophenone and z = hydrophobic portion of 2,5-dichlorobenzotrifluoride, o = 5.5, 7.1, 8.0, 9.9 and 13.2, respectively), showed outstanding chemical stability due to the ether (-o-)-free structure of the main chain. In addition, the SPPFPB-*o* membranes with IEC values ranging from 1.47 to 2.58 meq g^−1^ showed lower water absorption behavior compared to other hydrocarbon random copolymer-based PEMs due to the phase-separated architecture, with the ether-free structure of the main chain also contributing to this performance. Although the IEC values of the SPPFPB-*o* membranes were significantly larger than that of Nafion^®^ 212, the SPPFPB membranes (except for the SPPFPB-5.5 one) showed smaller dimensional change in the longitudinal direction due to the perpendicularly formed ion-conducting domains. The proton conductivities of the SPPFPB-5.5 and SPPFPB-7.1 membranes with higher IECs exhibited better proton conductivity than that of Nafion^®^ 212 under various operating conditions. Based on these results, including outstanding chemical stability, low dimensional change in the longitudinal direction and better proton conductivity than PFSA ionomer-based PEM, the authors claimed that the densely sulfonated poly(*p*-phenylene)-based copolymers are one of the best candidates for developing SHP-based PEMs with long-term durability while avoiding deterioration of proton conductivity. The PEM properties of the above-described copolymer-based PEMs including IEC, water uptake and proton conductivity are summarized in Table 5. Although most of the studies on densely sulfonated copolymer-based PEMs have been limited to focus on the polymer synthesis and their PEM properties, some of the studies reported the preliminary fuel cell performances of MEAs with the densely sulfonated copolymer-based PEMs including polarization curves and accelerated long-term stabilities at various operating conditions. These results indicated that the densely sulfonated copolymer-based PEMs are one of the possible candidates to instead of PFSA ionomer-based PEMs currently applied in mobile transportation and water electrolysis systems.

## 3. Conclusions

The development of sulfonated hydrocarbon polymer (SHP)-based PEMs has been actively pursued in order to overcome the inherent drawbacks of the PFSA ionomer-based PEMs. SHP-based PEMs composed of well-known aromatic random copolymers have shown relatively low proton-conducting behavior and physicochemical stability compared to PFSA ionomer-based PEMs due to the difficulty in forming the well-defined hydrophilic/hydrophobic phase-separated structures. To improve the proton conductivity of SHP-based PEMs without deterioration in physicochemical stability, control of the polymeric architecture is necessary to form distinct phase-separated structures between the hydrophilic and hydrophobic domains. Therefore, this review has focused on the synthetic procedures which underlie structure-engineered copolymers, such as block, graft/comb-shaped and densely sulfonated copolymers, and their PEM properties. Studies on SHP-based PEMs using block copolymers indicated that the length and structure of each hydrophilic and hydrophobic block can affect the size of ionic clusters as well as the phase-separation architecture, thereby affecting the PEM properties of the corresponding membranes. For graft/comb-shaped copolymers, degree, length and structure of side chains control the PEM properties such as proton conductivity and physical stability of the corresponding membranes. Finally, studies on densely sulfonated copolymers indicate that the degree of sulfonation of the hydrophilic reagents and location of the densely sulfonated moieties significantly affect nano-phase structures as well as the proton-conducting behavior of the resulting PEMs. Based on this review of existing literature, we strongly believe that, with rational designs of polymeric architecture beginning with the synthetic process, it will be possible to develop high-performance SHP-based PEMs surpassing the PFSA ionomer-based PEMs operating at harsh conditions of low RH. In addition, the exclusion of chemically vulnerable heteroatom linkages via the novel synthetic processes should be further considered to develop next-generation SHP-based PEMs for practical PEMFC applications.

## Figures and Tables

**Table 1:** Classification of fuel cells.

	AFC	MCFC	SOFC	PEMFC
Electrolyte	Aqueous solution of potassium hydroxide soaked in a matrix	Liquid solution of lithium, sodium and/or potassium carbonates, soaked in a matrix	Yttria stabilized zirconie	Solid organic polymer, poly-perfluorosulfonic acid
Fuel	Pure H_2_	H_2_, CO, CH_4_, other	H_2_, CO, CH_4_, other	Pure H_2_
Charge carrier	OH^−^	CO_3_^2−^	O^2−^	H^+^
Operating temperature	90–100 °C	600–700 °C	600–1000 °C	50–100 °C
Efficiency	60%	45–47%	35–43%	53–58%
Application	Military, Space	Electric utility, Large distributed generation	Auxiliary power, Electric utility, Large distributed generation	Backup power, Portable power, Small distributed generation, Transportation

**Table 2:** Representative structures of sulfonated hydrocarbon polymers used in polymer electrolyte membrane fuel cells.

Polymer	Structure
SPAES ^a^	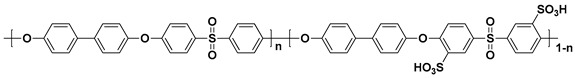
SPEEK ^b^	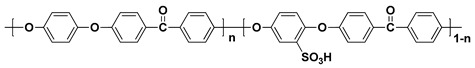
SPPO ^c^	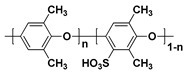
SPI ^d^	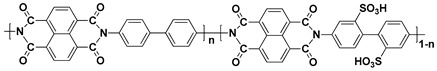

^a^ sulfonated poly(arylene ether sulfone); ^b^ sulfonated poly(ether ether ketone); ^c^ sulfonated poly(phenylene oxide); ^d^ sulfonated polyimide.

**Table 3:** Properties of block copolymer-based polymer electrolyte membranes.

Sample		IEC (meq g^−1^)	Water Uptake		Proton Conductivity		References
			Value (%)	Conditions (°C, % RH)	Value (mS cm^−1^)	Conditions (°C, % RH)	
SP3O-*b*-PAES-*b*-SP3O	X100	0.97	47.4	20, 100	9	90, 30	[63]
SPAE100-BPS0-HFB	5k–5k	1.30	35	rt, 100	50	30, 100	[64]
	10k–10k	1.38	68		100		
	15k–15k	1.40	79		110		
SPAE100-BPS0-DFBP	10k–5k	1.83	100		160		
	15k–10k	1.71	90		140		
Block-SPAES	X10Y10	2.90	390	rt, 100	480	80, 90	[67]
BPSH-*x*-PI-*x*	5–5	1.65	59	rt, 100	80	30, 100	[68]
	15–15	1.55	85		100		
	20–20	1.22	57		100		

**Table 4:** Properties of graft/comb-shaped copolymer-based polymer electrolyte membranes.

Sample		IEC (meq g^−1^)	Water Uptake		Proton Conductivity		References
			Value (%)	Conditions (°C, % RH)	Value (mS cm^−1^)	Conditions (°C, % RH)	
Mac-x	1a	0.87	20–25	80, 100	150	80–85, 100	[73]
	2a	1.40	45		430–450		
	3a	1.75	105		570–580		
SPPO	X5-Y6	0.92	28.2	rt, 100	150	rt, 100	[80]
	X5-Y9	1.28	52.3		190		
	X3-Y14	1.26	60.5		140		
	X5-Y14	1.27	75.6		210		
Comb-X	3	1.81	47.4	30, 100	195	80, 90	[49]
	7	2.06	107.1		213		
PSU-R	S1	1.83	24	30, 98	8	100, 50	[82]
	S4	1.96	29		10		
	S5	1.99	29		3		
	S6	2.23	37		20		

**Table 5:** Properties of densely sulfonated copolymer-based polymer electrolyte membranes.

Sample		IEC (meq g^−1^)	Water Uptake		Proton Conductivity		References
			Value (%)	Conditions (°C, % RH)	Value (%)	Conditions (°C, % RH)	
6*x*-SO_3_H	b	0.48	8.3	rt, 100	6.9	rt, 100	[92]
	c	0.47	7.8		3.7		
*X*-SO_3_H	18	1.09	43	rt, 100	91	rt, 100	[93,94]
	24	1.25	52		95		
8-SPES	17:83	1.80	25	80, 95	4–5	80, 50	[95,96]
	20:80	2.03	55		20–23		
		2.4	soluble	-	-	-	
10-SPES	13:87	1.96	40–43		10		
	15:85	2.21	50–53		15–20		
SPPFPB-*o*	5.5	2.58	71	rt, 100	258	70, 100	[99]
	8.0	2.08	48		170		
	13.2	1.47	27		95		
